# The Interdiffusion Behavior of NiCoCrAlYHf Coating Deposited by Arc Ion Plating on Carburized Ni-Based Single Crystal Superalloy

**DOI:** 10.3390/ma14237401

**Published:** 2021-12-02

**Authors:** Zun Chen, Jinyan Zhong, Shanglin Yang, Songmei Li, Jianhua Liu, Mei Yu

**Affiliations:** School of Materials Science and Engineering, Beihang University, Beijing 100191, China; chenzunzun@buaa.edu.cn (Z.C.); yslx001@buaa.edu.cn (S.Y.); songmei_li@buaa.edu.cn (S.L.); liujh@buaa.edu.cn (J.L.); yumei@buaa.edu.cn (M.Y.)

**Keywords:** superalloy, secondary reaction zone (SRZ), interdiffusion, HY5 coatings, carburization

## Abstract

In the present study, arc ion plating (AIP) was used to prepare a NiCoCrAlYHf coating (HY5 coating) on a carburized third-generation single-crystal superalloy DD10. The interdiffusion behavior of the carburized superalloy with an HY5 coating was investigated for a 1000 h oxidation time at 1100 °C. Carburization enhanced the interfacial bonding force and improved the microstructure of the NiCoCrAlYHf coating. An interdiffusion zone (IDZ) formed after a 300 h oxidation time, and the formation of a carburized layer effectively suppressed an inward diffusion of cobalt, aluminium, and chromium to the DD10 superalloy as well as an outward diffusion of nickel and refractory elements for instance rhenium and tungsten to the HY5 coating that occurred in static air at 1100 °C. The roles of the carburized layer in affecting thermal cyclic oxidation and element interdiffusion were studied. Subsequently, a modified form of the Boltzmann–Matano analysis was used to present the interdiffusion coefficients of aluminium.

## 1. Introduction

In the past decades, Ni-based single-crystal superalloys evolved for several generations for turbine blades of gas turbines and jet engines, which require thermal efficiency, oxidation resistance, and mechanical strength [1,2,3]. The third-generation superalloy by gradually adding refractory elements such as tungsten, molybdenum, and rhenium has good heat resistance, and it has high mechanical strength as well. However, the limitation of the melting points of single-crystal superalloys leads to the restriction of the rising service temperatures of single-crystal materials [4,5,6]. Nowadays, gas-turbine blades’ surface temperatures may attain 1150 °C or higher. In general, thermal barrier coatings (TBCs) are extensively applied for modern advanced turbine blades as thermal insulation coatings, which not only have good re sistance to excessive temperatures, but also may be subjected to hot corrosion [7,8,9]. Therefore, the thermal oxidation behaviors of advanced superalloys turn to be one of the vital factors of service life at such high temperatures.

A Ni-based single-crystal superalloy developed successfully these years, named DD10, containing additive refractory elements include tungsten, molybdenum, and rhenium, has been extensively explored as gas-turbine blades in advanced aero craft engines to improve their mechanical properties at high temperatures during work time. Nevertheless, these refractory elements accelerate the conformation of an unfavorable topologically close-packed phase (TCP) and a secondary reaction zone (SRZ) after long-time high-temperature exposure [10,11].

NiCoCrAlYHf coating, hereinafter referred to HY5 coating, is a metal-based coating. Due to its significant corrosion resistance and oxidation, HY5 coating services as a bond coat in a TBC system under high temperature. When the HY5 coating works in a long-term mega thermal environment, thermally grown oxide (TGO) forms owing to the kinetic and thermodynamics. NiCoCrAlYHf coating provides a TGO layer with sufficient aluminum. TGO effectively isolates the single-crystal superalloy from the aggressive environment, which remarkably leads to the reduction of the oxidation rate. Once the thickness of the TGO layer exceeds a crucial value, an irregular oxidation reaction would occur to consume the aluminum of the coating [12,13]. As the result of the inevitable elements’ interdiffusion between the substrate and the HY5 coating, the TCP phase and the SRZ would appear in the superalloy, both of which invade the TBC system’s performance and turn out to destroy the mechanical properties of the single-crystal superalloy [14,15,16,17]. Zhong et al. [18] have reported that a 70 μm-thickness SRZ, which consisted of needlelike or rodlike TCP phases, was taken shape after 300 h cyclic oxidation at 1100 °C in the DD10 single-crystal superalloy. The TCP phases were basically composed of refractory elements, for instance tungsten and rhenium, whereas the mass fraction of the aluminum was relatively low.

The SRZ formation gives rise to the deterioration of the mechanical properties of the single-crystal materials, and it is significant to come up with an effective method to dominate the SRZ formation in superalloys. Tin et al. [19] indicated that a 0.1 wt% carbon addition into a superalloy might promote the phase stability and restrain the TCP phases formation. Their study implies that penetrate carbon does have an advantage of inhibiting the degree of cellular transformation beneath the β-NiAl coating. Referring to their method, we applied the carburization technology to produce a carburized modified layer on DD10 to eliminate or suppress destructive TCP phases and SRZ formation, while maintaining the good adherence between the substrate and the HY5 coating.

To find out the mechanisms of carburization on limiting TCP phase and SRZ and element interdiffusion at 1100 °C during thermal cyclic oxidation, a 10 μm-thickness carburized layer was prepared by the technology of carburising-vacuum low-pressure diffusion on the DD10 superalloy. The morphology of the HY5 coating applied by the arc ion plating (AIP) method and the structure of the interface were observed for different thermal cycle times. The behavior of DD10 superalloy modified by carburization and the effect of this treatment on the interdiffusion behaviors of these materials under high temperatures were discussed.

## 2. Experimental Procedures

### 2.1. Substrate Material

The composition (in wt%) of DD10 was as follows: 4.0 Cr, 12.0 Co, 6.0 Al, 2.0 Mo, 6.0 W, 5.0 Re, and 7.0 Ta with a balance Ni matrix. The composition (in wt%) of HY5 was as follows: 18.0–23.0 Cr, 10.0–15.0 Co, 8.0–15.0 Al, 0.1–0.5 Y, and 0.2–0.6 Hf with a balance Ni matrix. The samples sizes were 30 mm × 10 mm × 1.5 mm. After ultrasonic cleaning in ethanol, the surfaces of thermal cyclic oxidation specimens were ground on 200#, 400#, 800#, and 1000# SiC paper.

### 2.2. Preparation of a Carburized Layer

**Figure 1:**
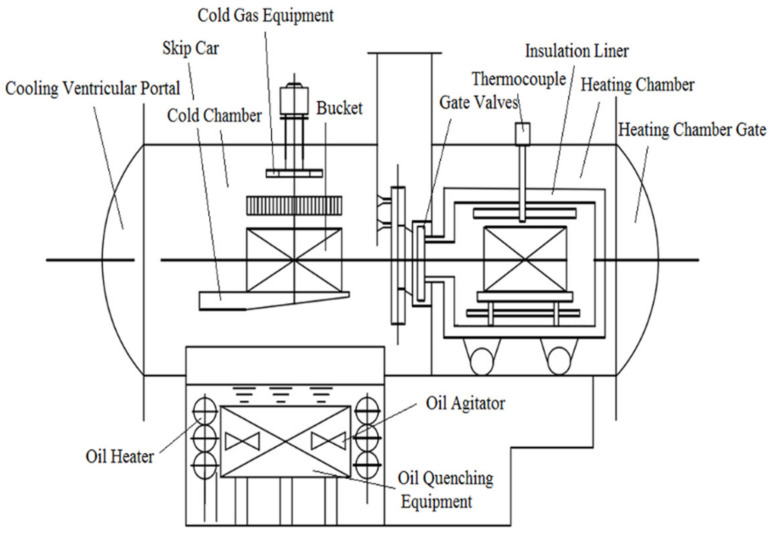
Sketch of the low-pressure carburizing vacuum chamber.

The surface of the substrate was mechanically polished and cleaned by ultrasound for 10 min, then put into a carburizing furnace and carburized after drying. The carburizing process was a low-pressure carburizing-vacuum diffusion-pulse-type process (Figure 1), while the carburizing agent was acetylene. The carrier gas was high-purity nitrogen. First, the surface of the substrate was pre-treated, and the pre-treatment temperature was at 920 °C. Secondly, in the low-pressure carburizing stage, the gas containing acetylene and nitrogen was introduced into the carburizing furnace at the pressure of 700–800 Pa, and the surface was kept under a high carbon potential for half an hour. Then, when the heating chamber was vacuumized to a high vacuum, the carbon atoms began to diffuse to the substrate surface. At an 800 A current, the carburizing agent acetylene was introduced, and the carburizing time was 3 h. The final rapid cooling was through high-purity nitrogen for about 2 min. The carburized layer thickness was about 10 μm.

### 2.3. Deposition of an HY5 Coating

A 35–50 μm-thickness HY5 coating was prepared by AIP (A-1000 Vacuum Arc Ion-Plating Unit) (HCSH-400, Guangzhou, China) at 1000 °C for 90 min. To enhance the interfacial bonding force between the substrate and the coating, the as-deposited specimens were annealed at 850 °C for 2 h in vacuum and finally, the typical morphologies of the coating were displayed. The preparation parameters of the HY5 coating were as follows: *U_b_*, 2 V; *I*_0_, 550–700 A; *p* < 6.67 × 10^−3^ Pa.

### 2.4. Thermal Cyclic Oxidation and Specimen Characterization

The carburized DD10 samples with an HY5 coating were studied in static air at 1100 °C for 300 h, 600 h, and 1000 h oxidation times separately, under a chamber pressure of 10^3^ Pa. An HY5 coating was deposited on all surfaces of the samples, including the sides. Each cycle included 55 min heating in the furnace and 5 min cooling out of the furnace in room temperature. An electronic balance (Sartorious BS 224S, Hamburg, Germany) was used to weigh the samples in a predetermined cycle with an accuracy of 10^−4^ g. The measured data were the average increase in weight of three parallel samples obtained by cyclic oxidation.

Using a solution containing HCl and HNO_3_ (volume ratio of 3:1) as the chemical etchant, dissolved selectively γ′ phase to facilitate the identification of precipitates in the microstructure. An X-ray diffractometer (D/MAX-2500, Tokyo, Japan), an optical microscope (OM), (LEICA DM4000, Wezlar, Germany), and a scanning electron microscope (CamScan3100, Cambridge, UK) equipped with an energy dispersive spectrometer (OXFORD X-Max) (CamScan3100, Cambridge, UK) were used to characterize the HY5 coating surface, interface morphology, and phase structure, respectively.

## 3. Results and Discussion 

### 3.1. The Morphology of the Carburized DD10 Superalloy with an HY5 Coating

Carburization is a progress that deposits a carburized layer permeating in a single-crystal alloy. The diffusion mechanism is applied to carburization, which can be explained that carbon diverts to the surface of the DD10 superalloy and generates a 10–20 μm thickness gradient of a compact layer as a carburized layer. The relationship between the substrate and the carburized layer is a metallurgical bond.

**Figure 2:**
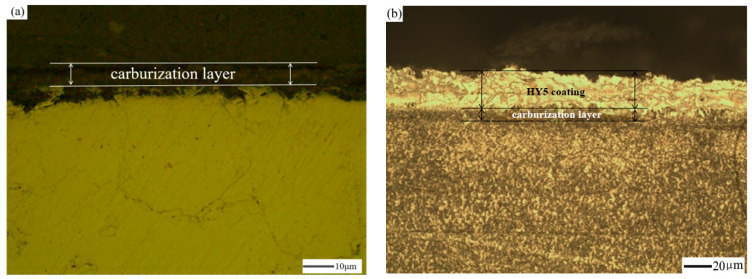
Metallography of the cross-sectional morphologies: (**a**) carburized Ni-based single-crystal superalloy; (**b**) carburized Ni-based single-crystal superalloy with an HY5 coating.

Figure 2a shows that the interface between the carburized DD10 single-crystal superalloy and the HY5 coating was not obvious. We can see in Figure 2b that the HY5 coating was intensive and uniform as well. This carburized layer helped maintain a good combination with the substrate DD10 superalloy. Therefore, carburization did not affect the microstructure of the DD10 superalloy deposited with an HY5 coating. Instead, it improved the microstructure slightly. The grain refinement of THE carburized superalloy with an HY5 coating improved the interfacial binding capacity and enhanced the service time of the TBC system. The reason why carburization ameliorated the morphology of the HY5 coating can be explained that the surface of the HY5 coating generates massive interstitial atoms, which reduced vacancy defects, when under the effect of high-energy particle bombardment. 

### 3.2. Thermal Cyclic Oxidation 

**Figure 3:**
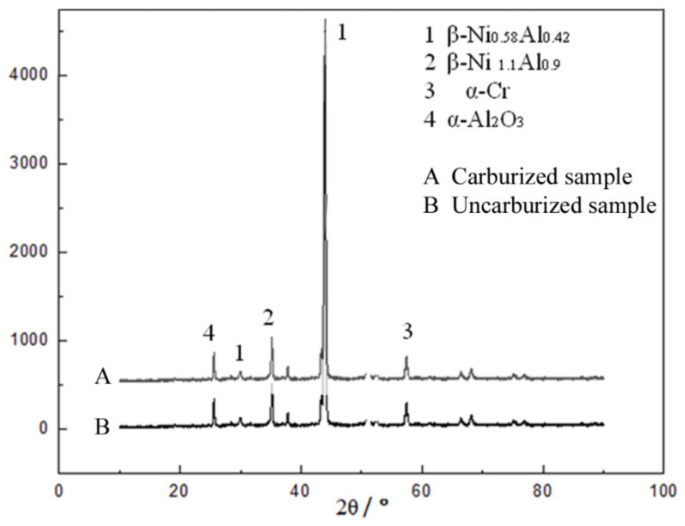
XRD patterns of the carburized and uncarburized DD10 superalloys with an HY5 coating after thermal cyclic oxidation at 1100 °C for 300 h.

XRD contrasting patterns in Figure 3 showed that the carburized DD10 superalloy with an HY5 coating had the same phases as the uncarburized DD10 superalloy with an HY5 coating after a 300 h thermal cyclic oxidation time, which consisted of β-NiAl, α-Al_2_O_3_, and α-Cr. These phases were proved to be a favorable condition to form a cubic-structure β-NiAl phase [20].

**Figure 4:**
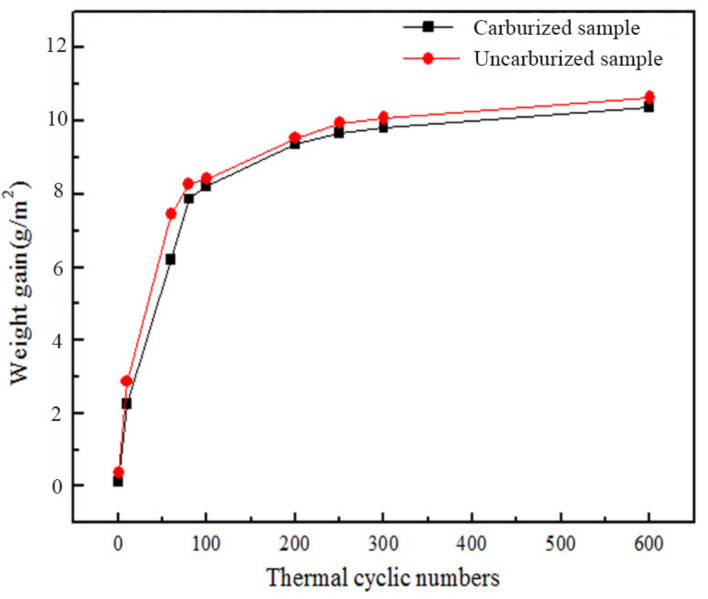
The kinetic curves of thermal cyclic oxidation of the carburized and uncarburized DD10 superalloys with an HY5 coating at 1100 °C.

The cyclic oxidation experiments of the uncarburized single-crystal superalloy DD10 and the substrate with an HY5 coating were performed for a 1000 h thermal cyclic oxidation time. It can be seen from Figure 4 that both the weights of the samples grew abruptly during the initial 80 h cyclic oxidation time and thereupon experienced a slower growth rate until finally becoming almost constant. It indicated that oxidation stage was steady and the thermal oxidation resistance of the superalloy DD10 after carburization treatment was desirable.

### 3.3. Morphology of the Carburized DD10 Superalloy with an HY5coating after Thermal Cyclic Oxidation

**Figure 5:**
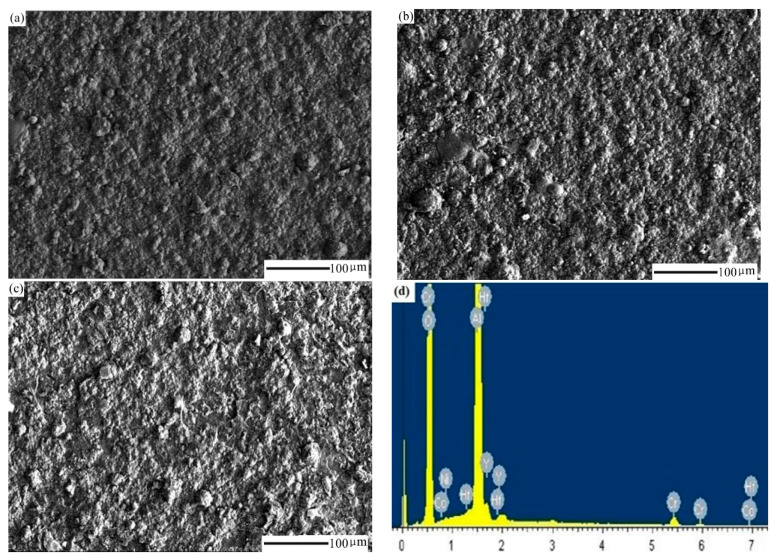
Backscatter-Electron (BSE) images of the surface morphologies of the carburized Ni-based single-crystal superalloy with an HY5 coatings after thermal cyclic oxidation at 1100 °C for different oxidation times: (**a**) 300 h; (**b**) 600 h; (**c**) 1000 h. (**d**) EDS spectra of (**a**).

As can be seen in Figure 5, the surface of the carburized DD10 superalloy with an HY5 coating consisted of compact tiny grains. An oxide film and a large number of relatively flat circular protrusion oxides were formed on the surface after 300 h oxidation (Figure 5a). As shown in Figure 5c, the size of grains was overwhelmingly larger after a 1000 h oxidation time, and the HY5 coating generated a quite compact oxide film that had no cracks and peeling on the surface. The surface of the HY5 coating primarily constituted Al_2_O_3_ and a very small amount of nickel and chromium by EDS analysis (Figure 5d). Due to the element diffusion, some elements existed on the surface of the HY5 coating, which were only contained in the substrate. An excess of the substrate elements such as tungsten, molybdenum, and chromium diffused to the oxide film on the surface of the coating. These elements formed volatile oxides, which formed tiny holes between the HY5 coating and the TGO to flake the oxide film. A very small amount of the substrate elements was detected in the surface of the HY5 coating in this experiment, which indicated that the carburized layer suppressed the elements diffusion between the HY5 coating and the substrate.

**Figure 6:**
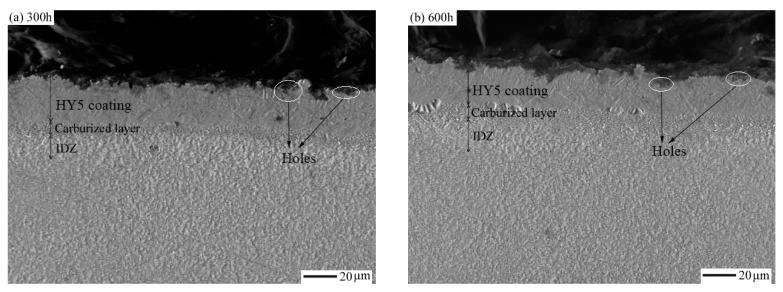

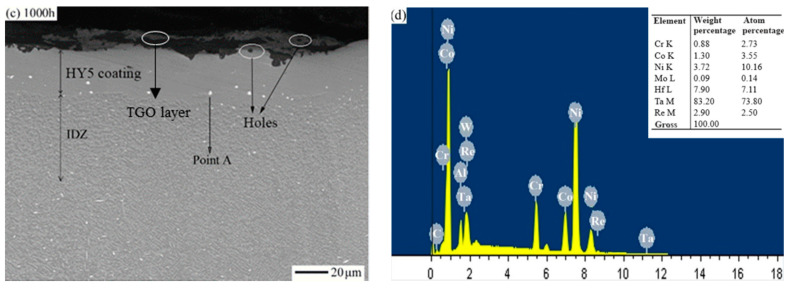
Backscatter-Electron (BSE) images of the cross-sectional morphologies of the carburized Ni-based single-crystal superalloy with HY5 coatings after thermal cyclic oxidation at 1100 °C for different oxidation times: (**a**) 300 h; (**b**) 600 h; (**c**) 1000 h. (**d**) EDS spectra of point A in (**c**).

Figure 6 shows the cross-sectional morphologies of the carburized DD10 single-crystal superalloy with an HY5 coating for 300 h, 600 h, and 1000 h thermal cyclic oxidation times at 1100 °C. The carburized layer, which was 8–10 μm, can be seen between the coating and the substrate in Figure 5a. With increasing thermal cyclic time, the HY5 coating gradually thinned. It is distinguished that a dark grey continuous film was a carburized layer with no white precipitate, as shown in Figure 6a. The carburized layer and the HY5 coating combined closely, and no holes and defects appeared after 300 h thermal cyclic oxidation. Fine granular carbide and α-Cr phase dispersed in the HY5 coating after 600 h and 1000 h oxidation times (Figure 6b,c). The TGO layer, which mainly consisted of α-Al_2_O_3_, became clear, complete and thick during thermal cyclic oxidation. The measured average thickness of the TGO layer did not exceed 10 μm. Meanwhile, the thickness of the HY5 coating mildly reduced. For a 1000 h oxidation time, the TGO spalled and became discontinuous [21].

An IDZ was observed in the specimen. Blocky carbides aggregated in the internal HY5 coating, and the formation of carbides separated the HY5 coating and the substrate to prevent the elements diffusion, which helped extend the life of the HY5 coating. There was no precipitate in the HY5 coating and the internal substrate after 300 h oxidation. A complete carburized layer still existed in the interface of the DD10 superalloy and the HY5 coating after 300 h, which was like a diffusion barrier layer. 

In the carburized DD10 superalloy with an HY5 coating after 600 h, no TCP phase was found, and the γ/γ′ structure maintained stable [22], demonstrating that the carburized layer acted as a diffusion barrier between the DD10 superalloy and the HY5 coating (Figure 6b). The carburized layer was invisible after 1000 h, as shown in Figure 6c, indicating that the carburized layer dissolved and the microstructure of the internal single-crystal superalloy changed with the oxidation time. Compared with for the 300 h oxidation time, the lattice roughened significantly for a 1000 h oxidation time. TCP precipitates were hardly observed after a 1000 h oxidation time at 1100 °C, and the morphology of the internal substrate did not change significantly, which demonstrated that the carburized Ni-based single crystal superalloy was protected approvingly by the high-tempeature oxidation-resistant HY5 coating (Figure 6c). The TCP precipitates were rich in rhenium, tungsten, chromium, and cobalt [18]. As seen in Figure 6c, small amounts of white round precipitates formed at the interface of the HY5 coating and the DD10 superalloy after 1000 h. The EDS analysis results of the white precipitates showed that the elements were mainly composed of carbon, nickel, cobalt, chromium, thallium, rhenium, and tungsten (Figure 6d). Therefore, MC carbides were suspected to precipitate from the HY5 coating and the carburized superalloy.

### 3.4. Interdiffusion Behavior between the HY5 Coating and the Carburized DD10 Superalloy 

**Figure 7:**
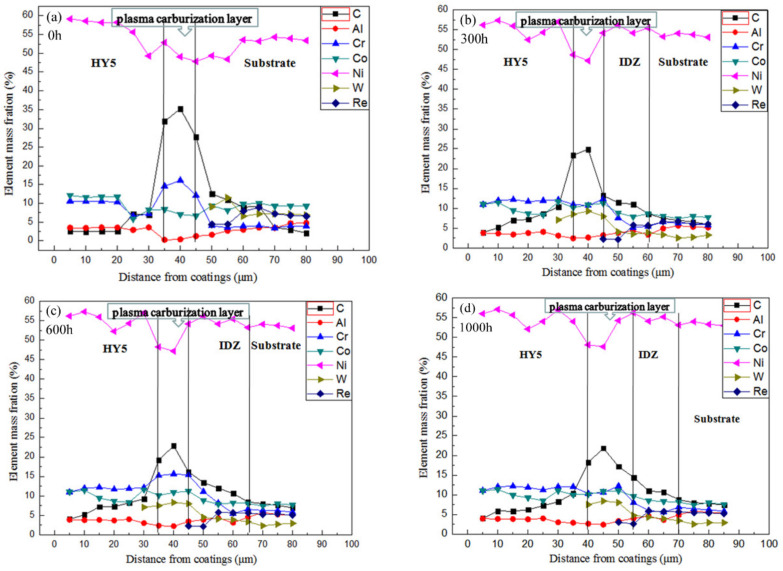
Chemical compositions of the carburized Ni-based single-crystal superalloy with an HY5 coating at 1100 °C after thermal cyclic oxidation: (**a**) 0 h; (**b**) 300 h; (**c**) 600 h; (**d**) 1000 h.

To observe the effect of the carburized layer on the interdiffusion behaviors of the HY5 coating, the cross-sectional composition profiles of the HY5 coating specimens were acquired by EDS, as shown in Figure 7. All the concentrations of the elements were steady in the initial deposited HY5 coating on the carburized DD10 superalloy. In particular, the concentrations of aluminium, chromium, and cobalt in the HY5 coating were intensely high (Figure 7a). Figure 7b presents that interdiffusion arose in the interface between the carburized DD10 superalloy and the HY5 coating, which was about 45 μm from the surface. The concentrations of aluminium, chromium, and cobalt in the HY5 coating decreased by cyclic time, as shown in Figure 7b–d. Due to the chemical gradient between the HY5 coating and the superalloy, aluminium, chromium, and cobalt diffused from the coating into the superalloy, while Ni, W, and Re diffused from the substrate to the HY5 coating. As illustrated in Figure 7, the concentrations of different elements changed slightly and remained basically steady. The carburized layer worked as a barrier layer to slow down the speeds of the elements interdiffusion. The HY5 coating degradation caused by interdiffusion was also effectively controlled.

The concentration of aluminium in the HY5 coating had a much higher level than that in the carburized DD10 substrate after 1000 h and reduced abruptly around the interface between the HY5 coating and the DD10 substrate. The concentration gradient of aluminium existed in both sides of the carburized layer, indicating that diffusion of the carburized layer had a significant impediment of aluminium. Carbon suppressed the segregation of aluminium in TGO and prevented pore from growing up in the interface of the TGO and the HY5 coating, which tended to significantly improve the adhesion of the TGO and the HY5 coating [12]. Meanwhile, the γ′ phase appearing inside the HY5 coating due to the formation of the TGO sustained the consumption of aluminium, while the diffusion of aluminium resulted in reducing the concentration of aluminium, which is the original β phase transition to the γ′ phase [18].

The concentration of aluminium in the HY5 coating reduced significantly after 1000 h and a 20 μm thickness IDZ formed between the coating and the substrate, demonstrated the occurrence of the interdiffusion between the uncarburized coating and the HY5 coating [18]. The interdiffusion behavior might alter the original chemical compositions in both the DD10 substrate and the HY5 coating, therefore giving rise to a decreased stability of the microstructure of the specimens and impacting the capacity of high-temperature corrosion and oxidation resistance as well [23].

The element diffusion occurred slightly between the carburized superalloy and the HY5 coating. Some elements diffused slightly, such as the concentrations of nickel and aluminium that displayed an abrupt change in the interface of the HY5 coating and the carburized superalloy. Besides, rhenium and tungsten were not detected in the HY5 coating after 1000 h, confirming that the carburized layer played a role of a diffusion barrier. The activity of aluminium played vital role in evaluating the tendency of samples to form an SRZ, and the diffusion coefficient of aluminium was calculated in this study [13,24]. 

Interdiffusion is element interpenetration due to the thermal motion; the direction is toward the decreasing of the carbon concentration to distribute evenly. It would be either self-diffusion of atoms or a foreign diffusion. 

Based on Arrhenius relationship and Fick’s second law [25], Matano [26] and Boltzmann [27,28] optimized a method to calculate diffusion coefficient *D* (called Boltzmann–Matano method). The basic principle of this method is that diffusion coefficient *D* is supposed as a function of concentration *C,* which can be written in the following form:

(1) $$D=-\frac{1}{2}\frac{\int_{0}^{C}xdC}{\frac{dC}{dx}}$$

$\frac{dC}{dx}$ is the slope of curve, $C=f\left(x\right)$ at the concentration of *C*; $\int_{0}^{C}xdC$ is the integral area of curve, $C=f\left(x\right)$ in the interval [0, *C*]; *D* is the diffusion coefficient at the concentration of *C*.

**Figure 8:**
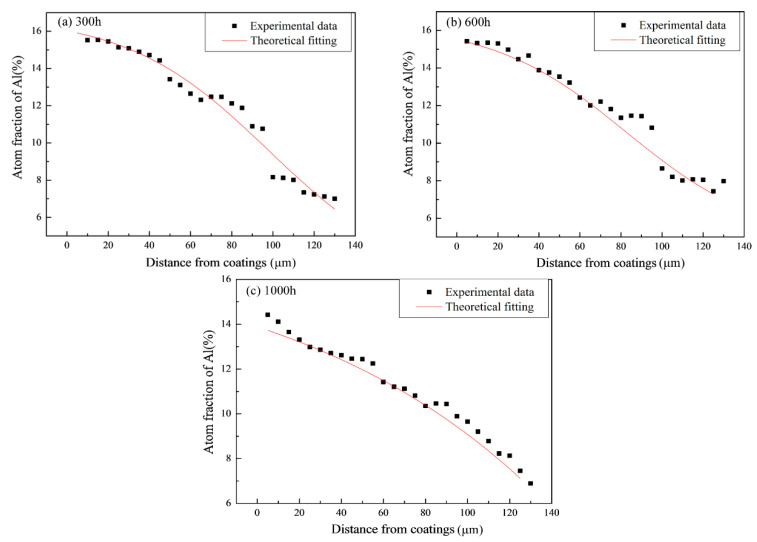
Concentration-fitted profiles of aluminum after thermal cyclic oxidation at 1100 °C: (**a**) 300 h; (**b**) 600 h; (**c**) 1000 h.

Figure 8 shows the concentration-fitted profiles of aluminum in different oxidation times at 1100 °C; the fitted curve results were consistent with those for the atomic concentration of diffusion coupled with changes of the distance.

**Figure 9:**
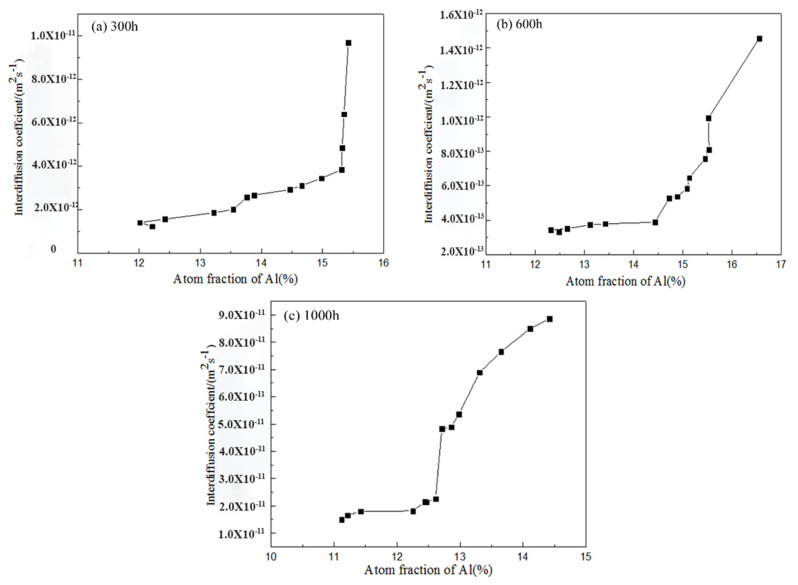
Various times of interdiffusion coefficients for aluminum with a variety of atomic contents: (**a**) 300 h; (**b**) 600 h; (**c**) 1000 h.

Using Equation (1) and fitting the curve equation (instead of the actual measured value), the diffusion coefficients of aluminum in different cyclic oxidation time at 1100 °C were calculated (Figure 9). Therefore, it appeared that diffusion of aluminum between the HY5 coating and the DD10 superalloy can be described by:

*D*_Al300_ = 4.51 × 10^−25^ exp(196.1*C*) (0.12 ≤ *C* ≤ 0.16),

*D*_Al600_ = 1.47 × 10^−24^ exp(82.64*C*) (0.12 ≤ *C* ≤ 0.17),

*D*_Al1000_ = 7.73 × 10^−20^ exp(135.7*C*) (0.11 ≤ *C* ≤ 0.15).

The results demonstrated that when temperature was constant, the diffusion coefficient increased significantly with the aluminum concentration in the HY5 coating from 300 h to 1000 h. The range was about five orders of magnitude. In addition, the aluminum diffusion between the HY5 coating and the DD10 superalloy in our previous work can be described by [18]:

*D*_Al300_ = 4.28 × 10^−24^ exp(184.8*C*) (0.11 ≤ *C* ≤ 0.16),

*D*_Al600_ = 1.09 × 10^−25^ exp(284.9*C*) (0.11 ≤ *C* ≤ 0.14),

*D*_Al1000_ = 5.69 × 10^−19^ exp(198.4*C*) (0.08 ≤ *C* ≤ 0.11).

The diffusion coefficient of aluminum was reduced relatively comparing experimental data between the carburized superalloy and the uncaburized superalloy, and the range was one order of magnitude [18]. By comparison of the carburized samples and the uncarburized samples, the aluminum diffusion coefficient reduced significantly at the same experimental temperature. The diffusion coefficient was closely related to chemical compositions and precipitates in the single-crystal superalloy.

The γ phase and the γ′ phase were found in the uncarburized samples; however, besides γ solid-solution phase, a variety of MC carbide precipitates were detected in the carburized single-crystal superalloy with an HY5 coating, which indicated that diffusion-controlled phase transformation occurred along with high-temperature oxidation. The diffusion-driving force enhanced in complex chemical compositions samples, not only due to the concentration gradient, but also the chemical potential gradient at high temperature [19,29,30].

Meanwhile, alloy elements in solid solutions changed the β-NiAl phase structure in the HY5 coating and chemical bonding properties. Solid-solution elements such as chromium, molybdenum, and cobalt may particularly reduce covalent fraction and increase metallic bond fraction among atoms in the β-NiAl phase, which increases the bonding force between atomics. Kim et al. [31] showed that lattice vacancies in β-NiAl phase have a great impact on interdiffusion while these alloy elements in solid solutions are likely to reduce its lattice vacancies, leading to a reduction in the diffusion coefficient.

If the temperature is constant, the diffusion of nickel and aluminum atomic solid phase is closely related to an effective diffusion path. As shown in Figure 6d, besides the β-NiAl, γ, and γ′ phases, a small amount of precipitated phases precipitated in the carburized single-crystal alloy with an HY5 coating. These white precipitates reduced the diffused cross-sectional area effectively to extend the diffusion path, and moreover, hindered the diffusion of aluminum, which lowered its diffusion coefficient.

### 3.5. Formation of an SRZ in the Carburized DD10 Superalloy with an HY5 Coating

An SRZ is generally observed under an IDZ of a bond coating in third-generation single-crystal superalloy containing much more refractory elements than the previous generations. In our previous work [18], an IDZ and an SRZ appeared in an uncarburized DD10 superalloy with an HY5 coating after 300 h cycling oxidation at 1100 °C. The SRZ consisting of white stripe and dot TCP phases was basically composed of refractory elements for instance rhenium and tungsten detected by EDS, whereas the mass fraction of aluminum was relatively low. It can be observed that the SRZ areas tended to grow in the internal place of the substrate, which caused a considerable mechanical properties degradation of the DD10 superalloy [18]. The Inward diffusion of aluminum and the outward diffusion of nickel leading to a stable γ/γ′ phase in the DD10 superalloy resulted in many refractory elements precipitating from the DD10 superalloy and the formation of an SRZ.

This will not only consume a large amount of solid solution strengthening elements, and the TCP phase as a brittle phase is the channel of crack formation and rapid crack propagation. Due to this effect, persistence, ductility, and toughness are seriously affected [16,17,32]. The morphology, distribution, and amount of TCP phase are usually the three ways to influence the mechanical properties superalloy [33]. However, a small amount of TCP has little impact on the performance of single-crystal superalloy.

In the present study, TCP phases were not observed under IDZ after 300 h after carburization, because some kinds of MC carbides generated around the carburized layer, so that α-Cr did not precipitate easily, which suppressed refractory elements rhenium and tungsten dissolved in α-Cr; thus, there was no TCP phase in the substrate. The γ′ phase was observed in the HY5 coating after 600 h due to the formation of TGO in Figure 6c. The formation of TGO needed to deplete aluminum. Meanwhile, aluminum transported from the HY5 coating via the IDZ to the substrate. The Al depletion by β-NiAl transition to the γ′-Ni_3_Al phase indicated that aluminum diffused inward from the HY5 coating. The amount of γ′-Ni_3_Al was much less than β-NiAl in the HY5 coating, suggesting the inhibition of inward aluminum diffusion by the carburized layer.

It should be noticed that an SRZ was not observed in the carburized samples even after 600 h thermal cyclic oxidation, which indicated that the carburized layer had a suppression effect on the formation of an SRZ. After 1000 h high-temperature oxidation, the TCP phase and the SRZ region were barely observed in DD10, indicating that the carburized layer effectively suppressed the diffusion between the DD10 superalloy and the HY5 coating.

The carburized layer served as a buffer layer and significantly reduced the rate of the interdiffusion between the DD10 superalloy and the HY5 coating before 1000 h oxidation. It was demonstrated that as compared to that in the uncarburized samples, the diffusion coefficient of aluminium in the carburized samples was decreased by about one order of magnitude. Carbon diffusing into the substrate formed the M_6_C phase and suppressed the outward diffusion of the superalloy. However, the diffusion rate of carbon in the β phase was much larger than that in the γ/γ′ phase. The carbon solid dissolved in the β phase, resulting in the dissolution of the M_6_C phase after 1000 h oxidation. Consequently, the carburized layer disappeared after 1000 h, and the content of carbon was reduced in the HY5 coating detected by EDS, which indicated that the inhibition of elements diffusion reduced at the same time. This showed that carbon cannot completely hinder interdiffusion but could slow down the inward aluminium diffusion and the outward substrate elements diffusion [34,35].

The M_6_C phase has a complex face-centered cubic structure. There are 96 metal atoms and 16 carbon atoms in a single cell. Metal atoms consist of large atoms of chromium, tungsten, and rhenium and small atoms of cobalt and nickel. They all have good solubility in the M_6_C phase. Rhenium, on the one hand, combined with chromium and cobalt, promotes the formation and stability of the M_6_C phase near a carburized layer [35,36,37]. This stability may inhibit the outward diffusion of elements chromium and tungsten at high temperature to protect mechanical properties of superalloy [38,39]. On the other hand, the formation of the M_6_C phase hinders the interdiffusion and delays the degradation of an HY5 coating. Furthermore, since the formation of M_6_C consumes most of the outward diffusion of carbon, tungsten, and a portion of cobalt, the tendency of formation of a brittle TCP phase reduces. 

As a result, the DD10 superalloy remained a stable γ/γ′ microstructure, and an SRZ did not occur even after 1000 h oxidation. As the interdiffusion of nickel and aluminium was validly restricted by the carburized layer, the microstructure of the DD10 superalloy was maintained and the strong TCP phase formed. For instance, rhenium and tungsten, were restrained in the M_6_C phase. As a result, the carburized layer, to some extent, improved the mechanical properties of the single-crystal superalloy. 

## 4. Conclusions

A NiCoCrAlYHf coating was deposited on a carburized third-generation single-crystal superalloy by AIP, and its properties after thermal cyclic oxidation were discussed. The study can be summarized as follows:

1. Carburization might enhance the interfacial bonding force and improve the microstructure of the NiCoCrAlYHf coating. The cyclic oxidation stage was steady, and the thermal oxidation resistance of the DD10 superalloy after carburization treatment was desirable after 1000 h.
2. The carburized layer, as a barrier at the interface of the single-crystal superalloy and the NiCoCrAlYHf coating, limited the aluminium inward diffusion, and therefore, restrained the γ/γ′ phase transformation in the superalloy during oxidation at 1100 °C. Consequently, the TCP phase, which was due to the dissolution of the refractory elements from the γ phase, was hindered effectively. The carburized layer effectively suppressed the refractory elements rhenium and tungsten from the outward diffusion from the substrate.
3. When the temperature was constant, the diffusion coefficient of aluminium increased significantly with the concentration of aluminium in the NiCoCrAlYHf coating after carburization. The range was five orders of magnitude. The diffusion of aluminium between the NiCoCrAlYHf coating and the carburized single-crystal superalloy can be described by:
  *D*_Al300_ = 4.51 × 10^−25^ exp(196.1*C*) (0.12 ≤ *C* ≤ 0.16),
  *D*_Al600_ = 1.47 × 10^−24^ exp(82.64*C*) (0.12 ≤ *C* ≤ 0.17),
  *D*_Al1000_ = 7.73 × 10^−20^ exp(135.7*C*) (0.11 ≤ *C* ≤ 0.15).
  Carburization treatment reduced the diffusion rate of aluminium by about one order of magnitude compared with that in previous study.
4. After 1000 h thermal cyclic oxidation, TGO had no obvious peeling, and merely a spot of the TCP phase and an SRZ were observed in the substrate, indicating that the carburized layer effectively suppressed the interdiffusion between the NiCoCrAlYHf coating and the DD10 single-crystal superalloy and the formation of an SRZ.
